# Prognostic value of National Early Warning Scores (NEWS2) and component physiology in hospitalised patients with COVID-19: a multicentre study

**DOI:** 10.1136/emermed-2020-210624

**Published:** 2022-03-14

**Authors:** Lauren J Scott, Alison Tavaré, Elizabeth M Hill, Lesley Jordan, Mark Juniper, Seema Srivastava, Emma Redfern, Hannah Little, Anne Pullyblank

**Affiliations:** 1 NIHR ARC West, University Hospitals Bristol NHS Foundation Trust, Bristol, UK; 2 Population Health Sciences, Bristol Medical School, University of Bristol, Bristol, UK; 3 West of England Academic Health Science Network, Bristol, UK; 4 Royal United Hospitals Bath NHS Foundation Trust, Bath, UK; 5 Great Western Hospitals NHS Foundation Trust, Swindon, UK; 6 North Bristol NHS Trust, Bristol, UK; 7 University Hospitals Bristol and Weston NHS Foundation Trust, Bristol, UK

**Keywords:** acute care, clinical assessment, clinical management, clinical care, COVID-19

## Abstract

**Background:**

National Early Warning Scores (NEWS2) are used to detect all-cause deterioration. While studies have looked at NEWS2, the use of virtual consultation and remote monitoring of patients with COVID-19 mean there is a need to know which physiological observations are important.

**Aim:**

To investigate the relationship between outcome and NEWS2, change in NEWS2 and component physiology in COVID-19 inpatients.

**Methods:**

A multi-centre retrospective study of electronically recorded, routinely collected physiological measurements between March and June 2020. First and maximum NEWS2, component scores and outcomes were recorded. Areas under the curve (AUCs) for 2-day, 7-day and 30-day mortality were calculated.

**Results:**

Of 1263 patients, 26% died, 7% were admitted to intensive care units (ICUs) before discharge and 67% were discharged without ICU. Of 1071 patients with initial NEWS2, most values were low: 50% NEWS2=0–2, 27% NEWS2=3–4, 14% NEWS2=5–6 and 9% NEWS2=7+. Maximum scores were: 14% NEWS2=0–2, 22% NEWS2=3–4, 17% NEWS2=5–6 and 47% NEWS2=7+. Higher first and maximum scores were predictive of mortality, ICU admission and longer length of stay. AUCs based on 2-day, 7-day, 30-day and any hospital mortality were 0.77 (95% CI 0.70 to 0.84), 0.70 (0.65 to 0.74), 0.65 (0.61 to 0.68) and 0.65 (0.61 to 0.68), respectively. The AUCs for 2-day mortality were 0.71 (0.65 to 0.77) for supplemental oxygen, 0.65 (0.56 to 0.73) oxygen saturation and 0.64 (0.56 to 0.73) respiratory rate.

**Conclusion:**

While respiratory parameters were most predictive, no individual parameter was as good as a full NEWS2, which is an acceptable predictor of short-term mortality in patients with COVID-19. This supports recommendation to use NEWS2 alongside clinical judgement to assess patients with COVID-19.

**Key messages**

**What is already known on this subject**

- National Early Warning Scores (NEWS2) are used to detect all-cause deterioration and improve outcomes in the general population.
- During the COVID-19 pandemic, it is not always possible to calculate a full NEWS2 due to remote consultation and management of patients on COVID-19 virtual wards.
- Patient physiology may not behave as anticipated in patients with COVID-19.

**What this study adds**

- In this retrospective multicentre study of over 1200 hospitalised patients with COVID-19, those with higher initial or maximum NEWS2 were more likely to die, require intensive care unit admission and have longer length of stay than patients with lower scores at either time.
- NEWS2 values had the best predictive value for short-term (2-day) mortality in patients with COVID-19.
- Individually, the respiratory components (respiratory rate, oxygen saturation and supplemental oxygen requirement) make the greatest contribution to the NEWS2 value.

## Introduction

The COVID-19 pandemic resulted in large numbers of patients presenting to hospital with acute respiratory failure. Early in the pandemic, it was recognised that despite hypoxaemia due to severe disease, some patients did not appear to manifest the usual symptoms of respiratory distress and the use of oximetry was promoted to assess oxygen saturation at presentation. In England, a pathway was introduced using oximetry measurement at home to monitor and detect deterioration in patients who did not require hospital admission.^1^

The National Early Warning Score (NEWS) was developed in 2012 by the Royal College of Physicians (RCP) to detect all-cause deterioration and improve outcomes in hospital patients.^2^ NEWS comprises respiratory rate (RR), oxygen saturation, temperature, systolic blood pressure (SBP), pulse and level of consciousness (alert, voice, pain, unresponsive). Each is scored 0–3 and combined to give an overall score with two additional points for supplemental oxygen. Scores range from 0 to 20, with more abnormal physiology resulting in higher scores. The updated NEWS2 has the addition of new onset confusion alongside level of consciousness and a new oxygen saturation scale (scale 2) for hypercapnic respiratory failure patients.^3^ NEWS2 is mandated by NHS England and NHS Improvement for use in acute hospital settings and the ambulance service and is recommended for use in out-of-hospital settings including general practice and community care.^2 4–7^

The NEWS2 calculation was included in the pathway for risk stratification of patients with COVID-19 assessed for management at home. There is evidence that NEWS2 is of benefit in out-of-hospital settings as it provides a common language of deterioration,^8–10^ but there has been debate regarding the value of NEWS2 in patients with COVID-19 outside hospital.^11^ During the COVID-19 pandemic, remote GP consultations and management of patients in virtual wards made recording NEWS2 difficult.^1^ There is therefore a need to understand which component parts of NEWS2 have value in identifying deterioration and poor outcome in patients with COVID-19.

Studies have examined the relationship of NEWS2 to outcome in patients with COVID-19. Almost all are single-centre studies involving small numbers, and many have focused on comparison of NEWS2 with other predictive scores. Unlike previous studies, our aim was to examine initial and maximum NEWS2 values and component scores in hospitalised patients with COVID-19 in the West-of-England. The aim was to determine if NEWS2 could be used to identify deterioration in patients with COVID-19 and then to clarify which, if any, of the individual parameters were most predictive of outcomes due to absolute value or change, to support clinical decision making.

## Methods

### Design and setting

This was a pragmatic, multicentre observational cohort study of COVID-19-positive patients admitted to North Bristol NHS Trust, University Hospitals Bristol and Weston NHS Foundation Trust, Great Western Hospitals NHS Foundation Trust and Royal United Hospitals Bath NHS Foundation Trust, in the West-of-England.

Patients were eligible if they were aged ≥16 years at admission and had a positive swab taken during their hospital stay or up to 2 weeks before. Patients admitted to hospital more than once following a positive test are described, but only first admissions were included in analyses.

### Data acquisition

Routinely collected electronic hospital data were requested from the four trusts. In each case, observations were measured manually but entered onto an electronic system, which recorded and displayed results. As data were routine and anonymised prior to transfer to the research team, patient consent was not required.

### Data

Data were requested on eligible patients from the first case in each trust (early March 2020) up until data extraction (approximately end of June 2020). As each trust extracted data at different times, data were amended to reflect the situation on 27 June 2020 (the date of first data extraction). All electronically recorded NEWS2 component physiological measurements were requested, along with date and time of each set of observations. Electronic observations were not recorded in the emergency departments (EDs) except for Great Western or the respiratory admission unit in Bath. NEWS2 is not used in intensive care units (ICUs).

Patient age, sex, hospital admission date, COVID-19 swab and result dates, ICU admission, hospital discharge status (hospital death, discharged, inpatient) and hospital discharge/death date were also requested.

### NEWS2 and component scores

Two NEWS2 values were considered for analysis. The initial score and its individual components were based on the first full set of electronically recorded observations taken within 2 days of the patient’s positive swab. Maximum NEWS2 values were the highest calculated NEWS2 values between initial NEWS2 and discharge/death for each patient. For some patients, the maximum score was the first score.

Maximum component scores were the highest component scores in this same period but were not always part of the maximum NEWS2.

Each component score (eg, temperature score of 0–3) and corresponding NEWS2 value were calculated from the physiological measurements listed above following the rules on the NEWS2 scoring card (online supplemental appendix 1).

10.1136/emermed-2020-210624.supp1 Supplementary data

NEWS2 change scores were calculated as maximum score minus first score; if a patient only had one score recorded, they were excluded from the change score analysis.

NEWS2 values were grouped into four categories for analysis: 0–2, 3–4, 5–6 and 7+, in line with hospital escalation trigger scores of 3, 5 and 7.^6^ When analysing change in NEWS2, a separate category of no change in NEWS2 was identified to recognise patients who did not deteriorate during admission.

### Outcomes

The primary outcomes were death in hospital with admission to ICU, death without ICU admission, ICU admission prior to discharge or no ICU admission prior to discharge. Outcomes for patients who were still inpatients when data were extracted are described but excluded from all analyses. Time to death for patients who died and post-COVID-19 length of hospital stay (LOS) for patients who survived to discharge were secondary outcomes. Time to death was calculated as date of death minus date of first score. LOS was calculated as discharge date minus COVID-19 swab date.

### Statistical analysis

Continuous data were summarised using medians and IQRs. Categorical data were summarised using counts and percentages.

The distribution of NEWS2 values and how this differed by mortality/ICU status were explored graphically. This analysis was split into three parts: first NEWS2 values, maximum NEWS2 values and the change from first to maximum. Similarly, distributions of each component score were explored. These analyses were purely descriptive.

The sensitivity and specificity of first NEWS2 at different cut-offs were determined for 2-day, 7-day, 30-day and any hospital mortality. Receiver operating characteristic curves (sensitivity against 1-specificity) were constructed and area under the curve (AUC) calculated along with 95% CIs. As prespecified, AUC values of 0.7–0.79 were considered acceptable, 0.8–0.89 excellent and ≥0.9 outstanding.^12^ Sensitivity and specificity were used to predict the positive and negative predictive values (post-test probabilities) of NEWS2 at cut-offs of 3, 5 and 7 to predict 2-day mortality.

Stata V.15.1 was used to conduct all data checking, cleaning and analyses.

## Results

### Demographics

**Figure 1:**
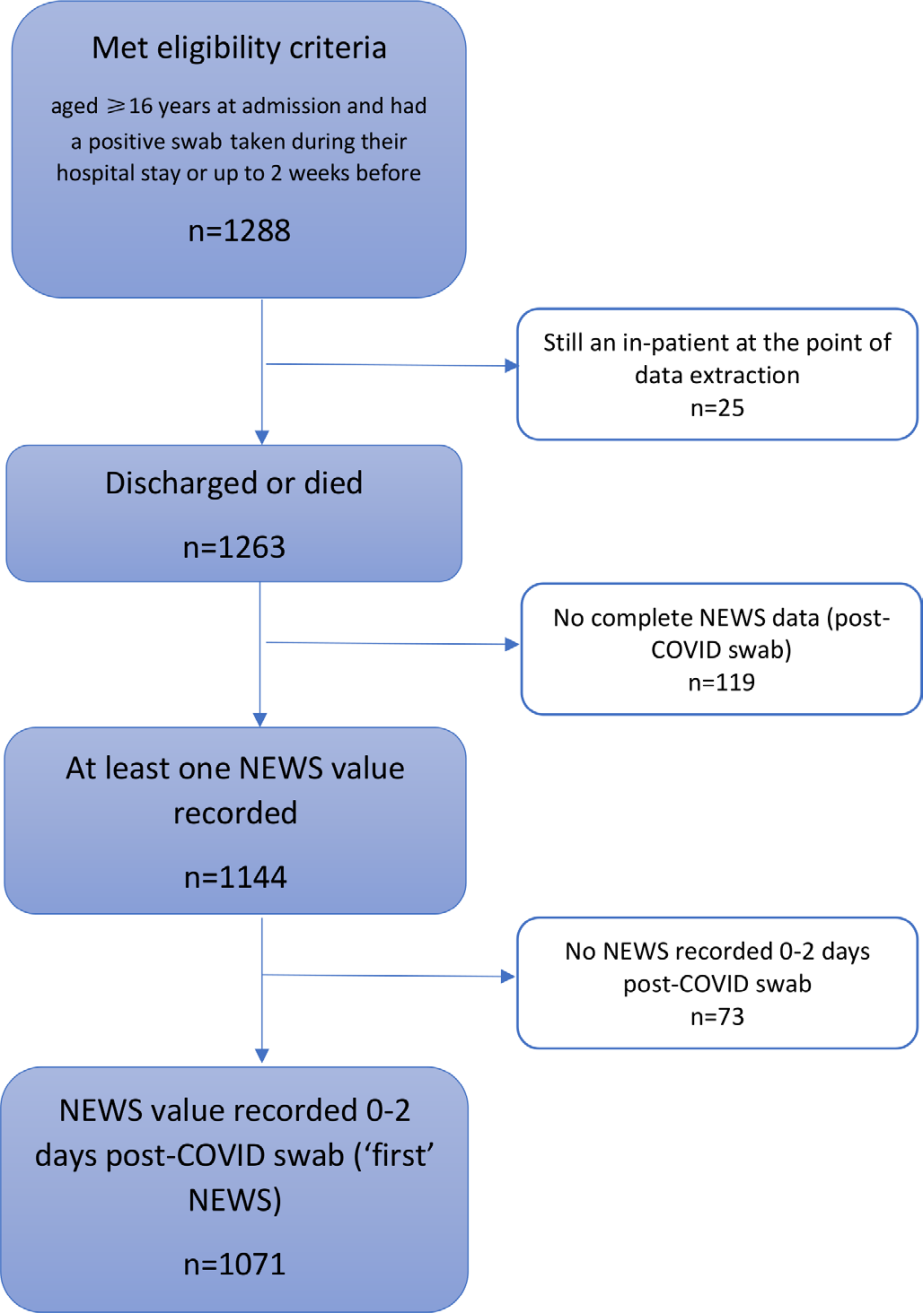
Flowchart of participants. NEWS, National Early Warning Score.

Between 11 March and 27 June 2020, 1288 hospitalised patients met the inclusion criteria (figure 1) of which 1263 had outcome data available and 1071 had a full NEWS2 recorded within 2 days of a positive COVID-19 swab. Five hundred and forty-one (42%) were women and median age was 74 years (IQR 59–84).

Three hundred and thirty-one (26%) died in hospital during their first admission, 40 of whom received ICU care before death. Eighty-three (7%) were admitted to ICU and subsequently discharged, and 849 (67%) were discharged without ICU requirement. Mortality was 4% at 2 days, 15% at 7 days and 26% at 30 days. Men were more likely to die than women (29% vs 23%), and older patients were more likely to die than younger patients (2%, 18%, 34% and 45% of patients aged 16–49, 50–69, 70–89 and 90+years, respectively).

Of those discharged from the hospital, 196/932 (21%) were readmitted (median time to first readmission 7 days, IQR 1–20); 147 were readmitted once, 31 twice and 18 three to six times. Twenty-six patients who were discharged following their first admission died during a subsequent admission. One thousand one hundred and forty-four (91%) patients had at least one set of complete electronic observations and 1071 (85%) had ‘first’ scores. Of the 618/1071 people who had their swab on the same day as they were admitted, 55% had their first NEWS2 recorded within 4 hours, 16% between 4 and 8 hours, 19% between 8 and 24 hours, and 10% over 24 hours later.

**Figure 2:**
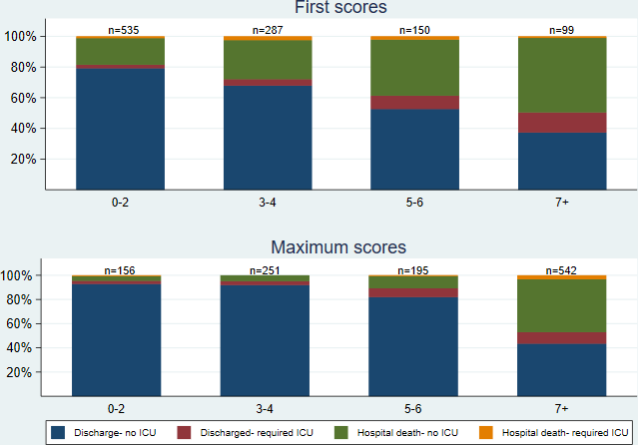
Outcome by first and maximum NEWS2. ICU, intensive care unit; NEWS, National Early Warning Scores.

Most patients had low first NEWS2 values: 535 (50%) NEWS2=0–2, 287 (27%) NEWS2=3–4, 150 (14%) NEWS2=5–6 and 99 (9%) NEWS2=7+. However, a greater proportion had a high score at some point during their stay: 156 (14%) had a maximum score of NEWS2=0–2, 251 (22%) NEWS2=3–4, 195 (17%) NEWS2=5–6 and 542 (47%) NEWS2=7+ (figure 2).

### Outcomes

**Figure 3:**
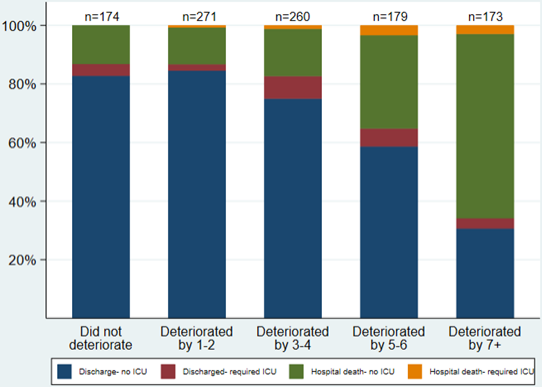
Outcome by change in NEWS2 from first to maximum. ICU, intensive care unit; NEWS, National Early Warning Scores.

Patients with higher first scores were more likely to die than patients with lower first scores (eg, 19% with first NEWS2=0–2 died compared with 49% with NEWS2=7+; figure 2); similarly, patients with higher maximum scores were more likely to die than those with lower maximum scores, although the effect was more pronounced: 4% with maximum NEWS2=0–2 died compared with 47% with NEWS2=7+ (figure 2). Only 16% of patients (174/1057) had no deterioration in NEWS2. Change scores had a similar distribution and relationship with mortality status as first scores (figure 3). Patients with larger change scores (ie, whose NEWS2 deteriorated more) were more likely to die; 13% who did not deteriorate, and 13% who only deteriorated by 1–2, died, compared with 66% who deteriorated by 7+. Patients with both high first NEWS2 and large change scores had the highest mortality (online supplemental file 4).

10.1136/emermed-2020-210624.supp4 Supplementary data

Patients with higher first NEWS2 were also likely to die earlier (online supplemental appendix 3). Among survivors, higher first and maximum NEWS2 values predicted longer LOS (online supplemental appendix 4).

10.1136/emermed-2020-210624.supp2 Supplementary data

10.1136/emermed-2020-210624.supp3 Supplementary data

### NEWS2 component scores

**Figure 4:**
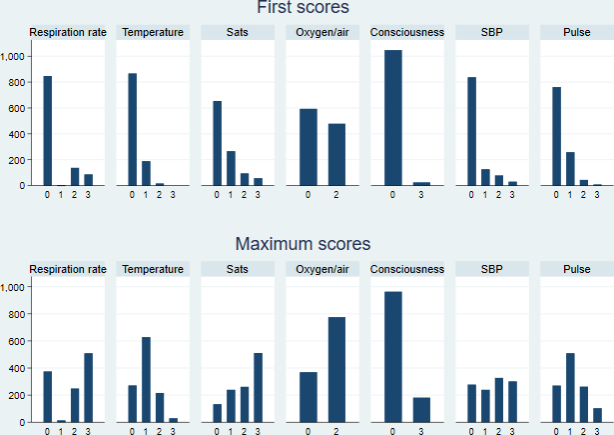
First and maximum component NEWS2. NEWS, National Early Warning Scores.

**Figure 5:**
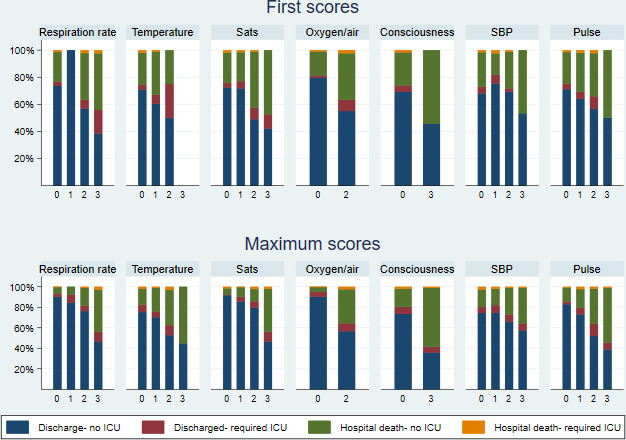
Outcome by first and maximum component NEWS2. ICU, intensive care unit; NEWS, National Early Warning Scores.

The components of first NEWS2 that most frequently had a value >0 were oxygen requirement (45% of patients), oxygen saturation (39% of patients) and pulse rate (29% of patients) (figure 4). For the maximum component scores, oxygen saturation scored >0 in 88% of patients; while most other components scored >0 in 67%–76% patients, except for consciousness which only ever scored >0 in 16% of patients (figure 4). In general, the higher the first or maximum component score, the higher the risk of death (figure 5). This relationship was less clear for first temperature and SBP scores.

We found 57/1071 first scores were 3 for low oxygen saturation, and of those 28/57 (49%) had a normal RR. This was more common for patients without supplemental oxygen (14/23, 61%) than with oxygen (14/34, 41%). Therefore, silent hypoxia was demonstrated in these patients, although numbers were small. This may be due to high percentage of patients receiving oxygen prior to first NEWS2 (45%).

### Sensitivity, specificity, AUC

**Figure 6:**
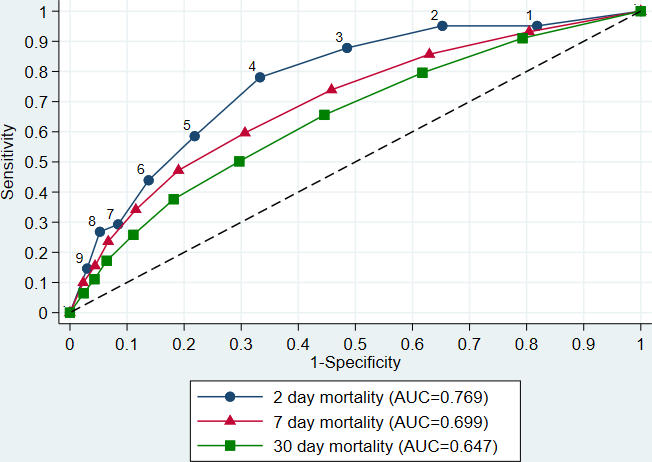
Receiver operating characteristic curve for 2-day, 7-day and 30-day mortality. AUC, area under the curve

The AUCs for 2-day, 7-day, 30-day and any hospital mortality were 0.77 (95% CI 0.70 to 0.84), 0.70 (0.65 to 0.74),0.65 (0.61 to 0.68) and 0.65 (0.61 to 0.68), respectively (figure 6). Two-day mortaility in this population (pre-test probability) was 3.8%. Using the most common NEWS2 cut-offs of 3, 5 and 7, post-test probabilities of 2-day mortality increased to 6.7%, 9.6% and 12.0%, respectively, for first scores ≥ the cut-off and decreased to 0.9%, 2.1% and 3.0%, respectively, for patients with first scores below the cut-off.

The AUCs for 2-day mortality for individual component scores were 0.71 (95% CI 0.65 to 0.77) for supplemental oxygen, 0.65 (0.56 to 0.73) oxygen saturation, 0.64 (0.56 to 0.73) RR, 0.56 (0.48 to 0.63) SBP, 0.53 (0.46 to 0.61) pulse, 0.53 (0.46 to 0.59) temperature and 0.53 (0.49 to 0.57) consciousness.

## Discussion

### Summary of results

This study included 1288 COVID-19-positive hospitalised patients in the West-of-England. Patients were more likely to be men and older, and these groups were also more likely to die, in line with other COVID-19 studies.^13–16^ Twenty-six per cent died during their initial admission and 2% died during a subsequent admission. Overall, 10% of patients were admitted to ICU. Most first NEWS2 values were low (50% NEWS2=0–2, 27% NEWS2=3–4), even though these patients were sick enough to be hospitalised. This is important when considering what threshold to use as an admission trigger in pre-hospital care and reinforces the advice that NEWS2 is an adjunct to clinical decision making and the need for admission should never be determined by NEWS2 value alone.^9 17^ However, 84% deteriorated with a worsening NEWS2 value and many patients subsequently had high scores at some point during their stay (17% maximum NEWS2=5–6, 47% maximum NEWS2=7+). Patients with higher first NEWS2 values were more likely to require ICU admission and/or die, in line with findings from non-COVID-19, mostly pre-hospital, populations.^8 18 19^ Only 7% of patients with maximum NEWS2=0–2 and 11% with maximum NEWS2=3–6 required ICU or died compared with 66% of patients with maximum NEWS2=7+. LOS for survivors increased as first and maximum NEWS2 increased.

The AUC for 2-day mortality was 0.77, reducing to 0.70 and 0.65 for 7-day and 30-day mortality; these AUCs were considered acceptable at 2 and 7 days according to our predefined criteria^12^ and support previous research suggesting that early warning scores are best at predicting short-term outcomes.^9 20^ An increase in most first and maximum component scores was associated with an increased risk of hospital mortality, although this relationship was less clear for temperature and SBP. For 2-day mortality, only oxygen requirement had an AUC which met the threshold for acceptable (0.71); oxygen saturation and RR were marginally predictive (AUC 0.65 and 0.64, respectively), but the other components alone were not predictive at all (AUCs 0.53–0.56).

### Strengths and limitations

The main strength of this paper is the inclusion of all patients with a positive COVID-19 diagnosis over a period of more than 3 months admitted to four hospitals. This enables findings to be generalisable across the UK. A limitation was the absence of electronic observations in ICU, three EDs and respiratory admission unit in Bath. This meant that 17% of patients were not included in the analysis of first scores, 11% were not included in maximum score analysis (figure 1), and maximum scores may not have been true maximums.

We would have liked to look at pre-hospital NEWS2, however without data linkage this would have meant studying suspected COVID-19 status rather than confirmed, and remote GP consultations mean that NEWS2 values in primary care data are often incomplete. We therefore focused on hospital data for confirmed patients with COVID-19. A limitation of this approach was that 45% of patients scored for supplemental oxygen on first score, which does not reflect the situation in primary care. However, it is likely that the score for supplemental oxygen would be replaced by a score for hypoxia in the community. We did not have access to admission symptoms, so it is possible that a proportion of the 402/1071 patients who had swabs taken at least 1 day post-admission were admitted with another diagnosis and acquired COVID-19 in hospital. However, this study was undertaken early in the pandemic when patients were not swabbed unless they had COVID-19 symptoms.

### Comparison with other literature

This is one of the largest UK multicentre studies of inpatients with confirmed COVID-19. In a systematic review of 18 studies with 6922 participants, only 6 had more than 400 patients.^17^ In the UK, the PRIEST study examined 20 891 suspected patients with COVID-19 in 70 EDs across UK.^21^ Six thousand five hundred and twenty-one were COVID-19 positive but not all of these were admitted. Other smaller UK studies^14 22–24^ and non-UK studies^25^ confirmed our finding that NEWS2 provides good prediction for adverse outcomes with a similar AUC^14 22 24^ and predicts a need for higher level care and not just mortality.^22 25^ NEWS2 compares well against other scores such as CURB-65 and q SOFA^14 21 22 26^ and confirms our finding that NEWS2 best predicts short-term mortality.^14^ Although NEWS2 predicts all-cause mortality, patients with COVID-19 have a higher mortality by NEWS2 compared with those with a non-COVID-19 diagnosis.^14^ In particular, a NEWS2>5 predicts an adverse outcome which aligns with a score of 5 being the trigger for escalation of care in patients with a non-COVID-19 diagnosis.^23^

Hypoxia (low oxygen saturation) has been shown to predict COVID-19 mortality in other studies.^16 26 27^ High RR has also been found to predict poor outcomes in this population.^28^ Concerns have emerged regarding ‘silent hypoxia’,^29^ but we have demonstrated that only 2.6% of patients (28/1071) had oxygen saturations ≤91% with a normal RR.

### Implications for research and/or practice

An evidence review of NEWS2 and COVID-19^11^ raised three research questions for the use of NEWS2 in primary care.

1. Is NEWS2 valid as a measure of severity in COVID-19, and does it predict who is likely to deteriorate? We have demonstrated that NEWS2 predicts mortality, particularly short-term mortality.
2. Is a single NEWS2 value sufficiently sensitive and specific? We have shown that a single score can predict short-term mortality, and based on the AUC, NEWS2=4 is the best value to balance sensitivity and specificity. A low score suggests that mortality is unlikely in the subsequent 2 days but over time, scores deteriorate in many patients, so the use of serial scores is likely to be superior to a single score. In either case, NEWS2 should always be used alongside clinical judgement and not as a rule in/out test.
3. Is calculating NEWS2 practical? Some components are measurable at home, but blood pressure and oxygen saturation require equipment. This study shows that blood pressure alone was a poor predictor of short-term mortality, but oxygen saturations and RR are most predictive. This supports the approach of using pulse oximeters for remote monitoring^5 30^ in COVID-19 virtual wards.

Further research measuring NEWS2 in patients with COVID-19 in primary/community care is required, but we believe the evidence presented in this study informs the management of patients in these settings, despite being collected in hospitalised patients. NEWS2 in combination with clinical judgement is a systematic way for clinicians to assess and manage patients according to the likelihood of deterioration and provides a standardised language to communicate illness severity.

This study has demonstrated that increased NEWS2 is associated with mortality in patients with COVID-19 and is a reasonably good predictor of 2-day mortality. The respiratory components (RR, oxygen saturation and supplemental oxygen requirement) are the most valuable predictors in the short-term supporting the use of pulse oximeters by COVID-19 oximetry@Home. These findings support the RCP’s recommendations to use NEWS2, alongside clinical judgement, in the assessment of patients with COVID-19.

## Data Availability

No data are available. N/A.
