# Supplementary figures and images for: Prognostic value of National Early Warning Scores (NEWS2) and component physiology in hospitalised patients with COVID-19: a multicentre study
Source: Emerg Med J. 2022 Mar 15;39(8):589–94. doi: 10.1136/emermed-2020-210624 (PMC8931800; doi:10.1136/emermed-2020-210624)

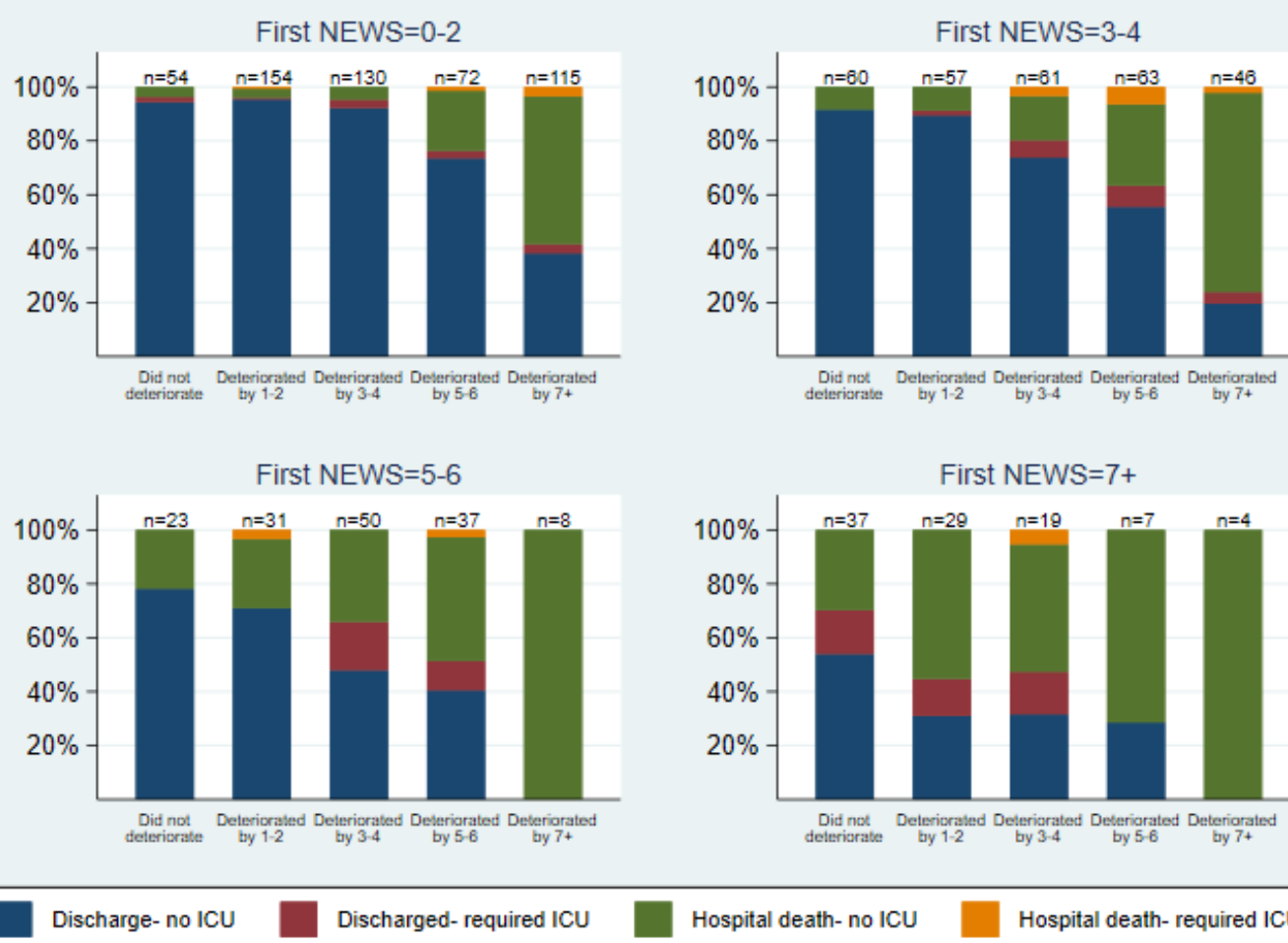

Supplement: Supplementary data [file emermed-2020-210624supp004.pdf]

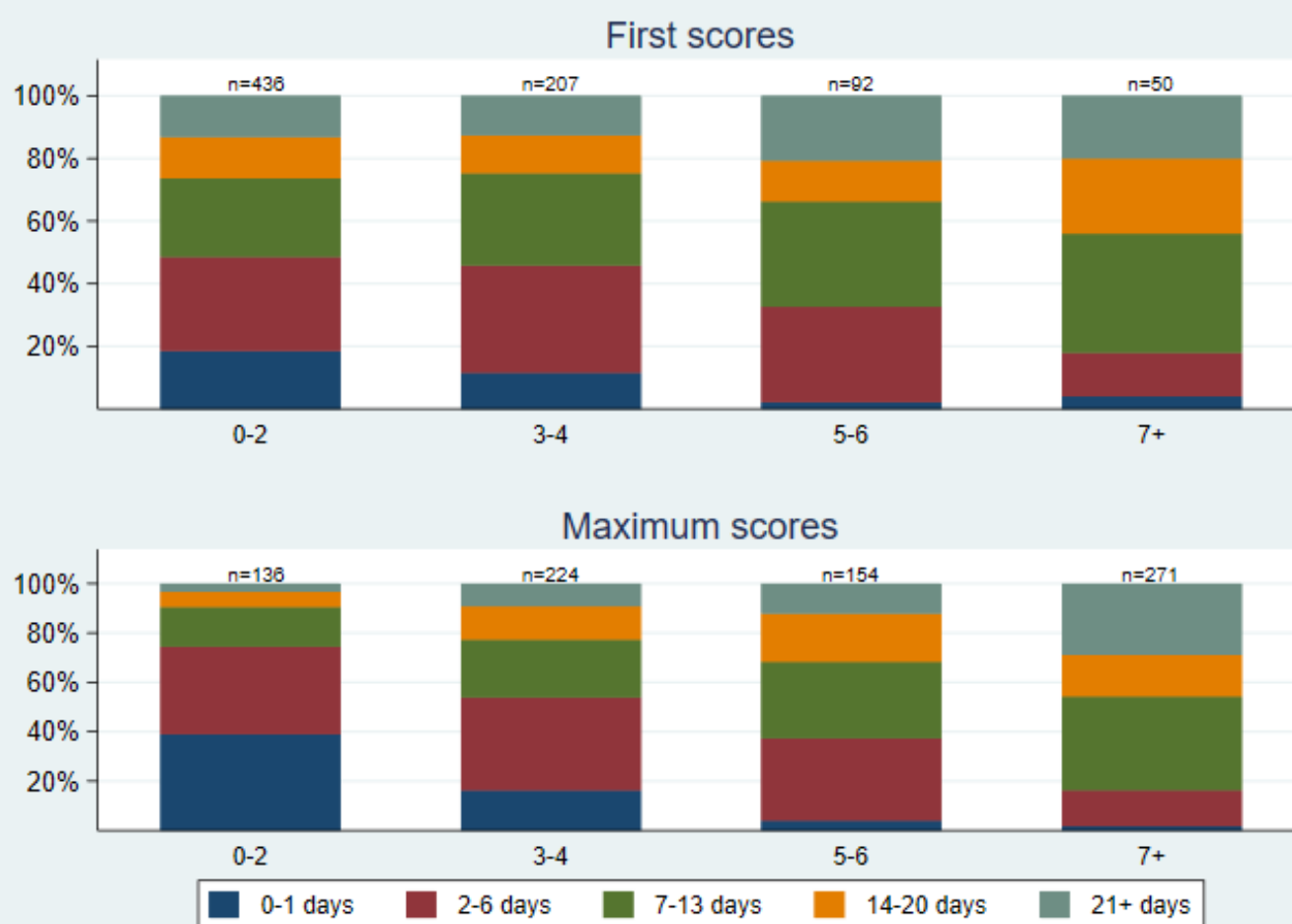

Supplement: Supplementary data [file emermed-2020-210624supp002.pdf]

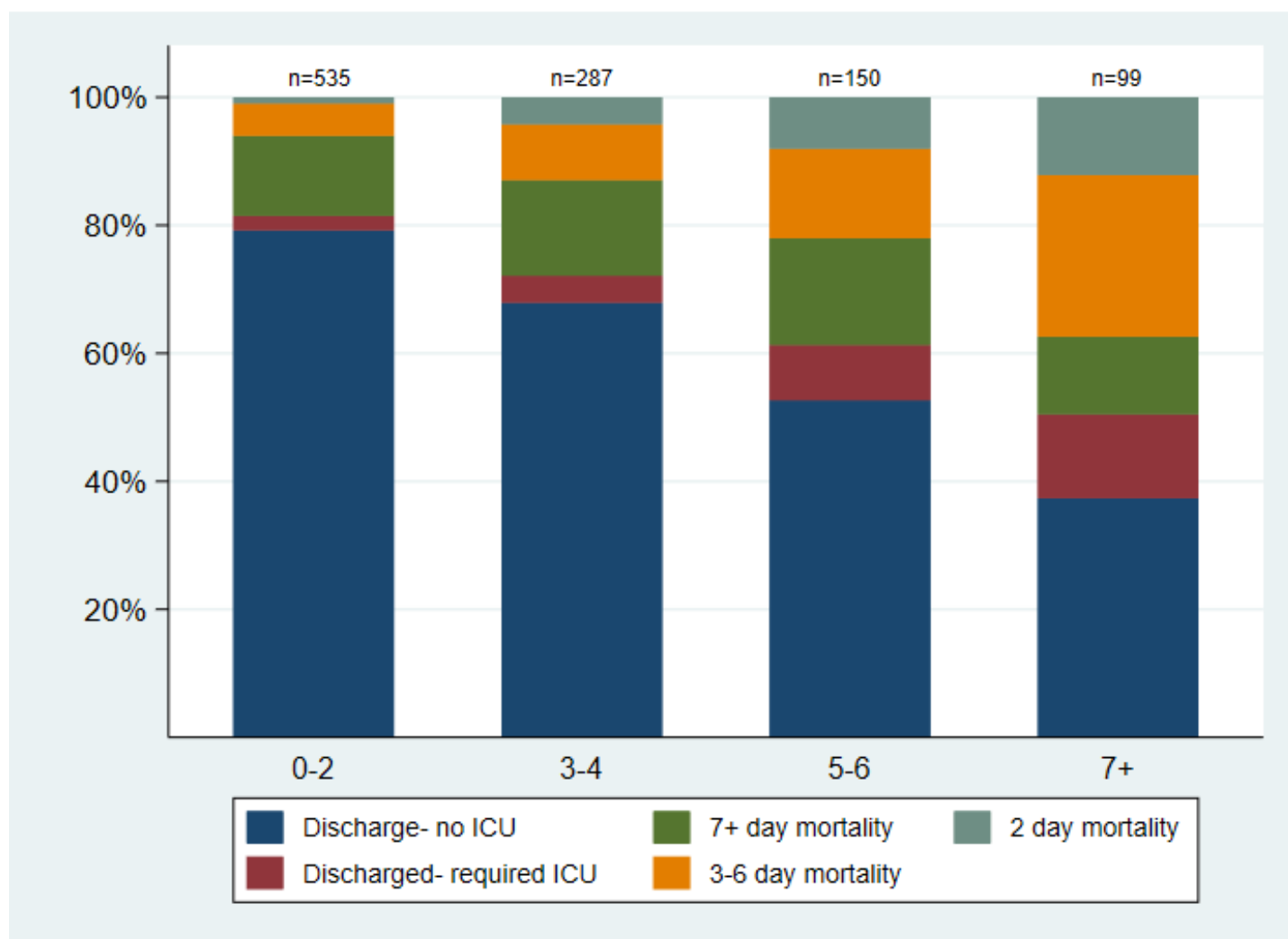

Supplement: Supplementary data [file emermed-2020-210624supp003.pdf]
