# Supplementary material for: Prognostic value of National Early Warning Scores (NEWS2) and component physiology in hospitalised patients with COVID-19: a multicentre study
Source: Emerg Med J. 2022 Mar 14:emermed-2020-210624. doi: 10.1136/emermed-2020-210624 (PMC8931800; doi:10.1136/emermed-2020-210624)
Supplement: Supplementary file 1 [file emermed-2020-210624supp001.pdf]

**Physiological parameter**	**Score**						
	**3**	**2**	**1**	**0**	**1**	**2**	**3**
Respiration rate (per minute)	≤8		9–11	12–20		21–24	≥25
SpO_2 Scale 1 (%)	≤91	92–93	94–95	≥96			
SpO_2 Scale 2 (%)	≤83	84–85	86–87	88–92<br>≥93 on air	93–94 on oxygen	95–96 on oxygen	≥97 on oxygen
Air or oxygen?		Oxygen		Air			
Systolic blood pressure (mmHg)	≤90	91–100	101–110	111–219			≥220
Pulse (per minute)	≤40		41–50	51–90	91–110	111–130	≥131
Consciousness				Alert			CVPU
Temperature (°C)	≤35.0		35.1–36.0	36.1–38.0	38.1–39.0	≥39.1	
