# Supplementary material for: Prognostic value of National Early Warning Scores (NEWS2) and component physiology in hospitalised patients with COVID-19: a multicentre study
Source: Emerg Med J. 2022 Mar 14:emermed-2020-210624. doi: 10.1136/emermed-2020-210624 (PMC8931800; doi:10.1136/emermed-2020-210624)
Supplement: Supplementary file 2 [file emermed-2020-210624supp002.pdf]

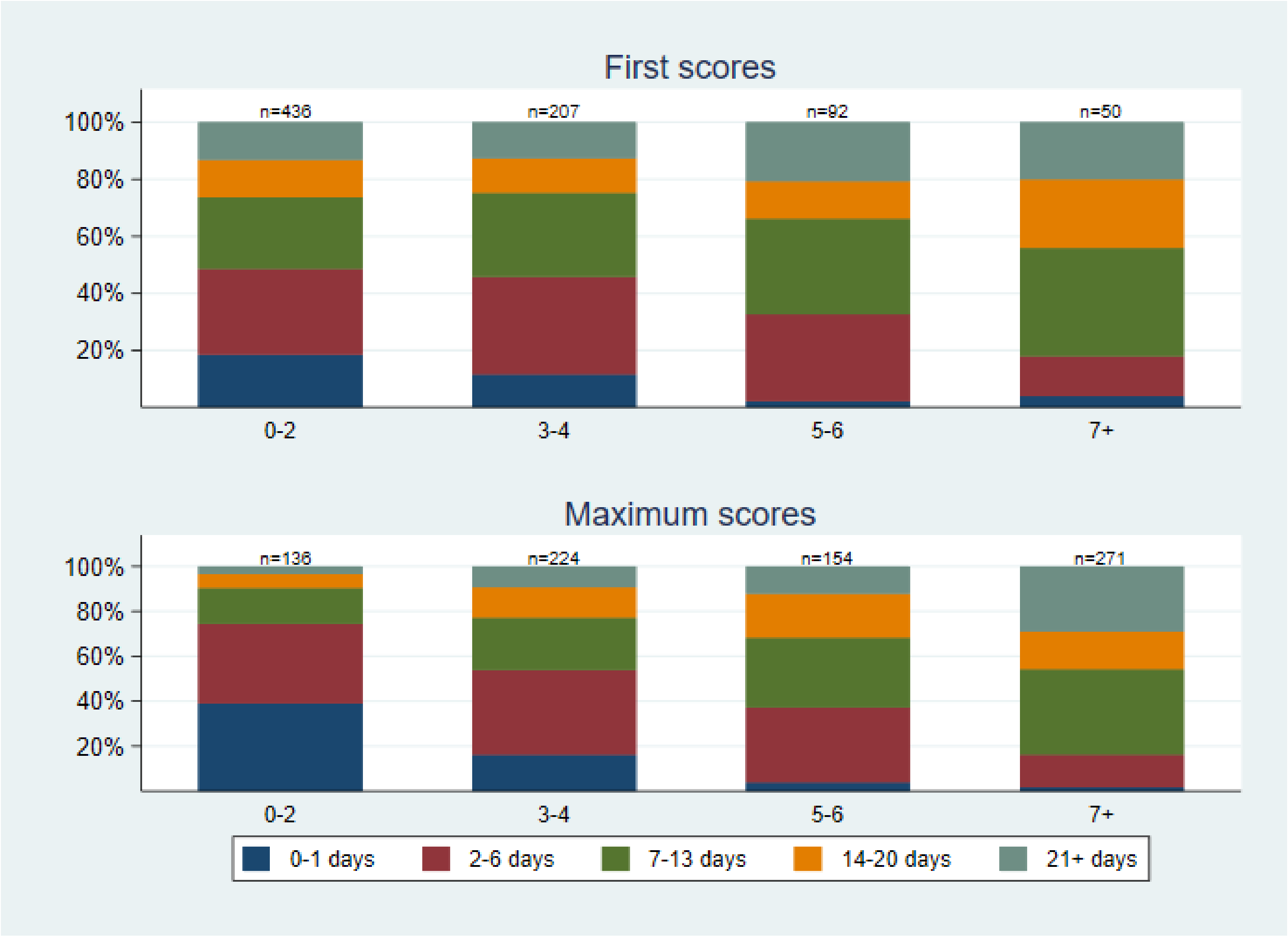
First scores
100%
80%
60%
40%
20%
n=436
n=207
n=92
n=50
0-2
3-4
5-6
7+
Maximum scores
n=136
n=224
n=154
n=271
0-1 days
2-6 days
7-13 days
14-20 days
21+ days
