# Supplementary material for: Prognostic value of National Early Warning Scores (NEWS2) and component physiology in hospitalised patients with COVID-19: a multicentre study
Source: Emerg Med J. 2022 Mar 14:emermed-2020-210624. doi: 10.1136/emermed-2020-210624 (PMC8931800; doi:10.1136/emermed-2020-210624)
Supplement: Supplementary file 3 [file emermed-2020-210624supp003.pdf]

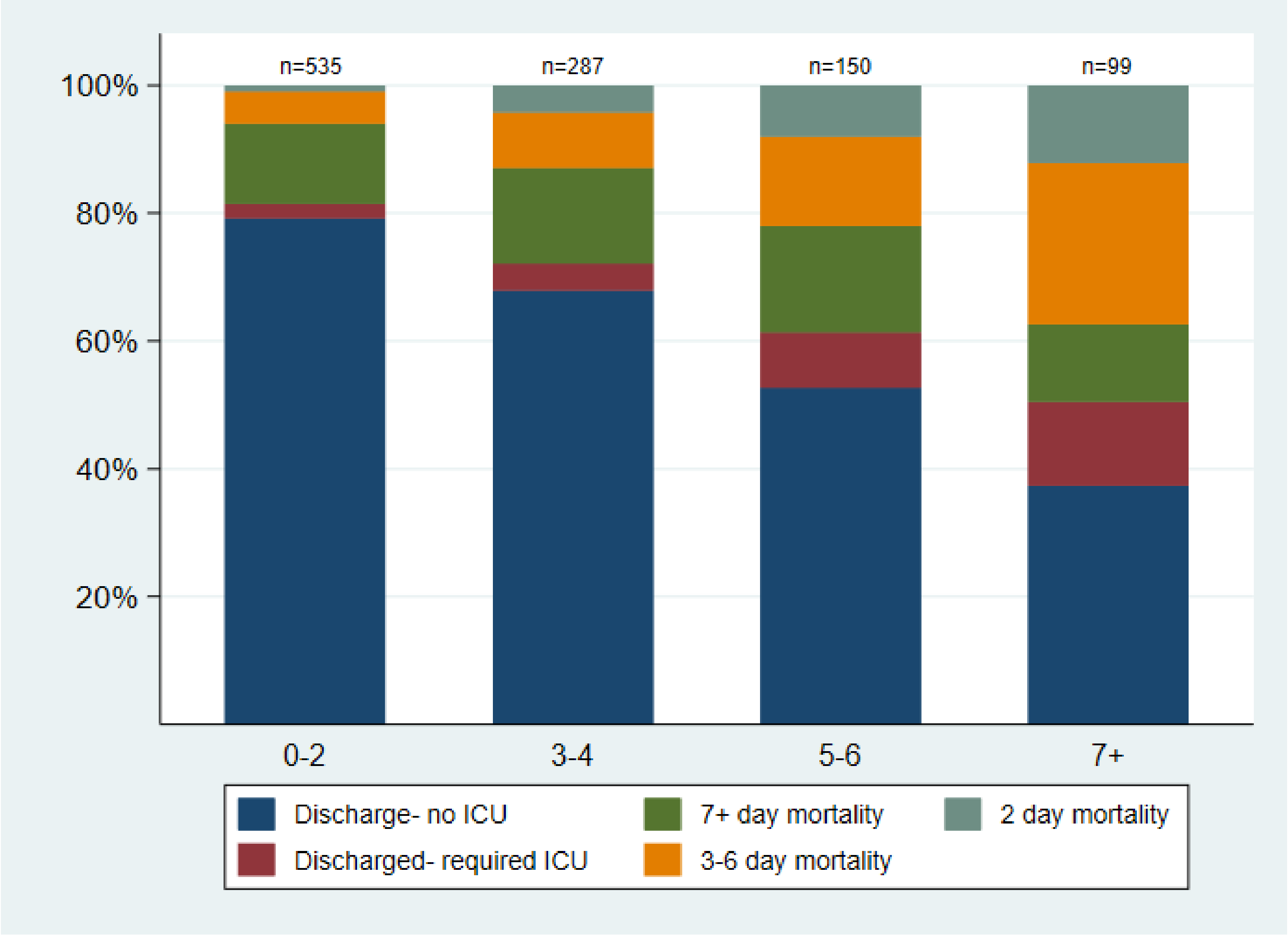
n=535
n=287
n=150
n=99
100%
80%
60%
40%
20%
0-2
3-4
5-6
7+
Discharge- no ICU
Discharged- required ICU
7+ day mortality
3-6 day mortality
2 day mortality
