# Supplementary material for: Prognostic value of National Early Warning Scores (NEWS2) and component physiology in hospitalised patients with COVID-19: a multicentre study
Source: Emerg Med J. 2022 Mar 14:emermed-2020-210624. doi: 10.1136/emermed-2020-210624 (PMC8931800; doi:10.1136/emermed-2020-210624)
Supplement: Supplementary file 4 [file emermed-2020-210624supp004.pdf]

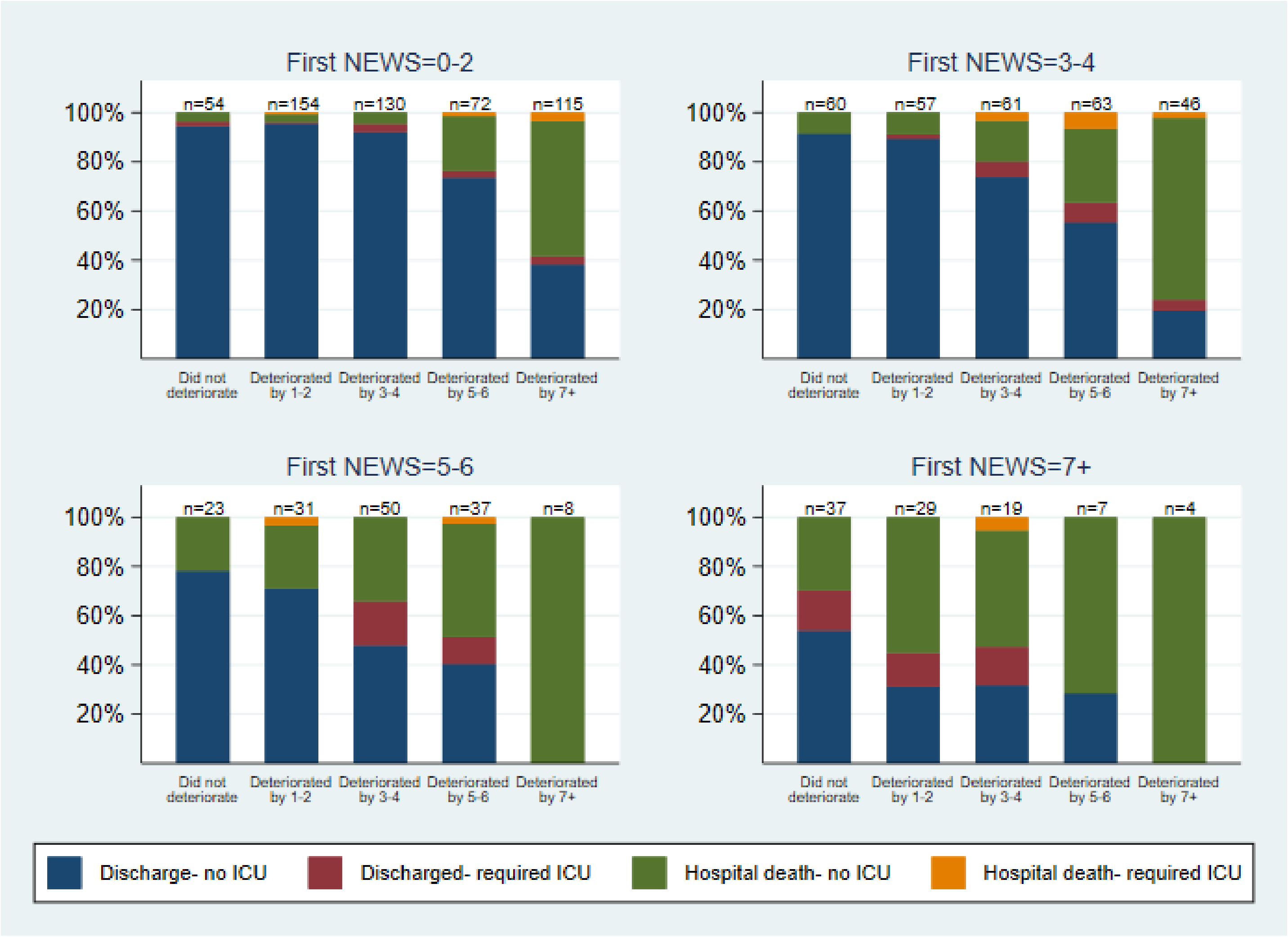
First NEWS=0-2
n=54
n=154
n=130
n=72
n=115
First NEWS=3-4
n=60
n=57
n=61
n=63
n=46
First NEWS=5-6
n=23
n=31
n=50
n=37
n=8
First NEWS=7+
n=37
n=29
n=19
n=7
n=4
100%
80%
60%
40%
20%
Did not deteriorate
Deteriorated by 1-2
Deteriorated by 3-4
Deteriorated by 5-6
Deteriorated by 7+
Discharge- no ICU
Discharged- required ICU
Hospital death- no ICU
Hospital death- required ICU
